# The Effects of a Gate Bias Condition on 1.2 kV SiC MOSFETs during Irradiating Gamma-Radiation

**DOI:** 10.3390/mi15040496

**Published:** 2024-04-04

**Authors:** Chaeyun Kim, Hyowon Yoon, Yeongeun Park, Sangyeob Kim, Gyuhyeok Kang, Dong-Seok Kim, Ogyun Seok

**Affiliations:** 1 Department of Electronic Engineering, Kumoh National Institute of Technology, Gumi-si 39177, Republic of Korea; cykim@kumoh.ac.kr; 2 Department of Semiconductor System Engineering, Kumoh National Institute of Technology, Gumi-si 39177, Republic of Korea; hwyoon@kumoh.ac.kr (H.Y.); yepark@kumoh.ac.kr (Y.P.); syk@kumoh.ac.kr (S.K.); ghkang@kumoh.ac.kr (G.K.); 3 Korea Atomic Energy Research Institute, Gyeongju-si 38180, Republic of Korea; dongseokkim@kaeri.re.kr; 4 School of Electronic Engineering, Kumoh National Institute of Technology, Gumi-si 39177, Republic of Korea

**Keywords:** SiC MOSFET, gamma-ray, radiation, total ionizing dose, power device

## Abstract

We investigated the effects of gate bias regarding the degradation of electrical characteristics during gamma irradiation. Moreover, we observed the punch through failure of 1.2 kV rated commercial Silicon Carbide (SiC) Metal Oxide Semiconductor Field Effect Transistors (MOSFETs) due to the influence of gate bias. In addition, the threshold voltage (V_T_) and on-resistance (R_on_) of the SiC MOSFETs decreased significantly by the influence of gate bias during gamma irradiation. We extracted the concentration of carriers and fixed charge (Q_F_) in oxide using N-type SiC MOS capacitors and Transmission Line Measurement (TLM) patterns to analyze the effects of gamma irradiation. The Total Ionizing Dose (TID) effect caused by high-energy gamma-ray irradiation resulted in an increase in the concentration of holes and Q_F_ in both SiC and oxide. To analyze the phenomenon for increment of hole concentration in the device under gate bias, we extracted the subthreshold swing of SiC MOSFETs and verified the origin of TID effects accelerated by the gate bias. The Q_F_ and doping concentration of p-well values extracted from the experiments were used in TCAD simulations (version 2022.03) of the planar SiC MOSFET. As a result of analyzing the energy band diagram at the channel region of 1.2 kV SiC MOSFETs, it was verified that punch-through can occur in 1.2 kV SiC MOSFETs when the gate bias is applied, as the TID effect is accelerated by the gate bias.

## 1. Introduction

Silicon Carbide (SiC) Metal Oxide Semiconductor Field Effect Transistors (MOSFETs) are attracting attention in high-voltage space applications owing to superior characteristics of SiC [1,2,3,4]. These advantages make them suitable for designing power semiconductors for space environments. However, exposure to gamma-ray increases the positive fixed charge (Q_F_) in the oxide, leading to deterioration of electrical and material properties [5,6,7].

When gamma-ray irradiates the MOS structure, Electron–Hole Pairs (EHPs) are generated in the oxide due to the high energy of the gamma-ray. As a result, holes remain in the oxide due to the relatively higher carrier mobility of electrons compared to that of holes. Total Ionizing Dose (TID) effects occur in the SiC region and oxide, and cause changes in electrical characteristics such as the threshold voltage (V_T_) and on-resistance (R_on_) of MOSFETs [8,9,10].

Several studies have analyzed changes in the electrical characteristics of commercially available silicon and SiC power MOSFETs due to radiation exposure [11,12,13]. The results suggest that TID effects on power MOSFETs mainly result in changes in I-V characteristics, especially a decrease in V_T_ and an increase in current drive [14,15,16].

Gamma irradiation causes defects at the interface of the SiO_2_/SiC, which reduces channel mobility and increases leakage current [17,18,19,20]. In the space environment, since the gate voltage is applied to the SiC MOSFET in the turn-on state, it is necessary to consider the gate voltage conditions when analyzing the effects of gamma radiation on the SiC MOSFETs. However, the effects of gate bias conditions on SiC MOSFETs exposed to gamma radiation have been reported by several research groups but require further investigations.

In this study, we analyzed the influences of TID effects on electrical characteristics of 1.2 kV SiC MOSFETs according to gate bias conditions. The commercial device used for measurement and analysis is the 1.2 kV rated SiC MOSFET from STMicroelectronics (model number: SCTWA20N120). We fabricated P^+^ Transmission Line Measurement (TLM) patterns and N-type MOS capacitors in order to analyze the factors causing variations in the electrical characteristics of the 1.2 kV SiC MOSFET according to dose of gamma-ray and gate bias conditions. We observed that the TID effect occurs not only in the SiC region but also in the oxide of 1.2 kV SiC MOSFETs. In addition, we discovered that the TID effect is accelerated in devices where the gate voltage is applied and elucidated the mechanism by which the breakdown voltage (BV) of 1.2 kV SiC MOSFETs decreases through TCAD simulations.

## 2. Experimental Results

In our experiments, we performed static current-voltage (I-V) measurements of a commercial 1.2 kV/189 mΩ N-channel SiC MOSFET. We also analyzed the N-type SiC MOS capacitor Test-Elemental Groups (TEGs) and Transmission Line Measurement (TLM) that we fabricated for the analysis of the degradation mechanism. The devices were irradiated with gamma-radiation from a Co-60 source at the Advanced Radiation Technology Institute (ARTI) of Korea Atomic Energy Research Institute (KAERI). Each device was exposed to gamma-ray doses of 1000, 5000, and 10,000 Gy. The commercial SiC MOSFETs were measured with Keysight’s B1505A (Power Device Analyzer, Keysight, Santa Rosa, CA, USA), and the N-type SiC MOS capacitor and P^+^ TLM were measured with Keysight’s B1500A (Semiconductor Device Parameter Analyzer, Keysight, Santa Rosa, CA, USA). Table 1 summarizes the experimental conditions and bias conditions on the SiC MOSFETs and SiC MOS capacitors used in the experiment.

**Figure 1:**
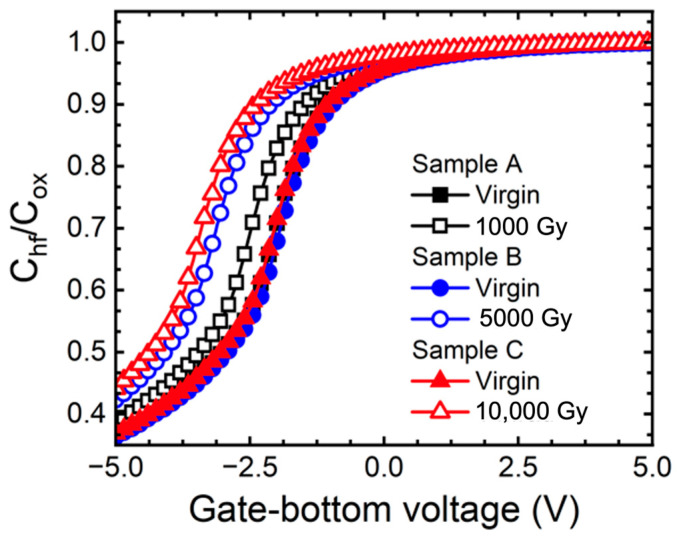
Measured C-V curves at 1 MHz (C_hf_) normalized to oxide capacitance C_ox_ for N-type SiC MOS capacitors with floating state for different gamma ray doses (1000, 5000, and 10,000 Gy).

**Figure 2:**
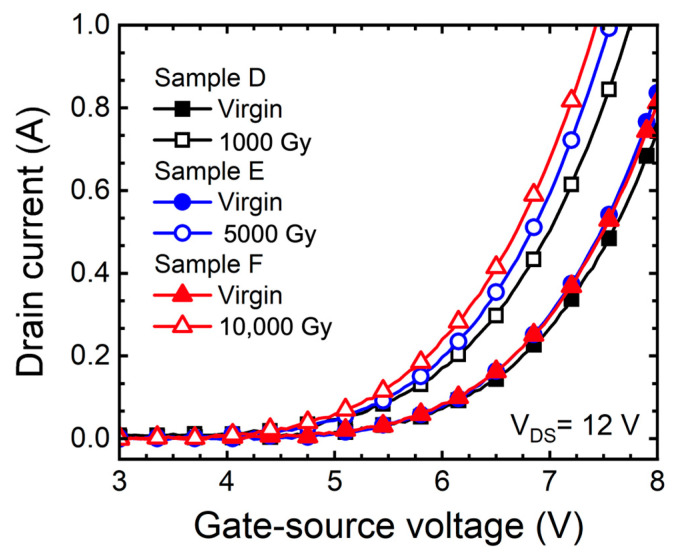
Measured transfer curves (I_D_-V_GS_) of 1.2 kV SiC MOSFETs with floating state according to gamma-ray irradiation dose (1000, 5000, and 10,000 Gy).

Figure 1 shows the C-V characteristic of the MOS capacitor depending on the doses of gamma-ray. C_hf_ is the value of the capacitance measured at a frequency of 1 MHz and C_ox_ is the value of the capacitance of the oxide. The fixed charge in the oxide was extracted to verify the TID effects as a deterioration mechanism for the change in electrical characteristics of the 1.2 kV SiC MOSFETs. The shift of the flat band voltage (ΔV_FB_ = V_FB,ideal_ − V_FB,measured_) and the shift of fixed charge in the oxide (ΔQ_F_ = Q_F,before_ − Q_F,after_) were defined [21]. Table 2 summarizes the extracted the ΔV_FB_ of the MOS capacitor and the Q_F_ as the doses of gamma-ray increases. We realized that as the gamma-ray increased, the Q_F_ increased by 0.59 × 10^11^ cm^−2^ (sample A), 1.70 × 10^12^ cm^−2^ (sample B), and 1.94 × 10^12^ cm^−2^ (sample C). When the MOS structure was irradiated with a high-energy gamma-ray, EHPs were generated within the oxide. In addition, defects were created in the oxide due to the effects of gamma irradiation, and holes were easily trapped in this region [18]. As a result, it was noticed that the Q_F_ of the MOS capacitor increased depending on the doses of gamma-ray [5,6,10,22,23]. Figure 2 depicts the transfer curve (I_D_-V_GS_) of the SiC MOSFETs in floating state depending on the doses of gamma-ray. The V_T_ was extracted to verify the change in characteristics of the 1.2 kV SiC MOSFETs according to gamma-ray irradiation.

Figure 2 depicts the transfer curve (I_D_-V_GS_) of the SiC MOSFETs in floating state depending on the doses of gamma-ray. The V_T_ was extracted to verify the change in characteristics of the 1.2 kV SiC MOSFETs according to gamma-ray irradiation.

As a result, as the doses of gamma-ray increased, the shift of V_T_ (ΔV_T_ = V_T,before_ − V_T,after_) is 0.25 V (sample D), 0.45 V (sample E), and 0.55 V (sample F). The decrease in V_T_ caused by hole traps created by the TID effects. The increase in Q_F_ affects the charge distribution on the SiC surface and affects the reduction of V_T_ [10,15,24,25].

**Figure 3:**
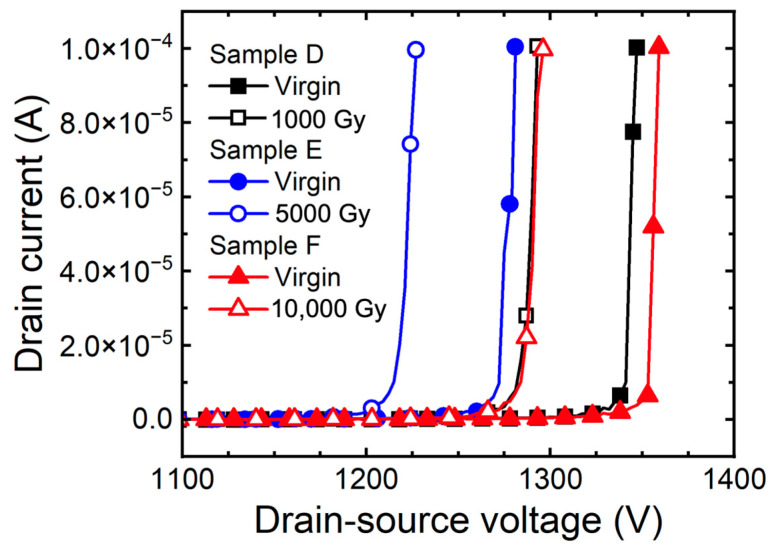
Measured breakdown characteristic curves (I_D_-V_DS_) of 1.2 kV SiC MOSFETs with floating state according to gamma-ray irradiation dose (1000, 5000, and 10,000 Gy).

Figure 3 shows the measured breakdown characteristic curve (I_D_-V_DS_) according to the gamma-ray irradiation doses of 1.2 kV SiC MOSFETs without bias. The shift of in breakdown voltage (ΔBV = BV_before_ − BV_after_) as the doses of gamma-ray increased to 1000 Gy, 5000 Gy, and 10,000 Gy is 52 V (sample D), 54 V (sample E), and 63 V (sample F). Hole traps are generated in the thick oxide at the edge termination region induced by the TID effects, affecting reduction of the surface depletion and the electric field at the surface, thus reducing the BV [21].

As the V_T_ decreases, unintended turn-on may occur, leakage current may increase, and breakdown characteristics are deteriorated, resulting in power loss or deterioration of reliability. To analyze the phenomenon from numerous aspects, P^+^ TLM and SiC N-type MOS capacitor-TEG were used in the experiment. The concentration of P^+^ TLM according to gamma-ray irradiation was extracted [21]. We extracted sheet resistance through P^+^ TLM and analyzed by extracting concentration of holes. As a result of extracting the term in which the mobility of the hole and the concentration are multiplied according to concentration, the concentration of P^+^ increased by 2.89% and 3.12% as the doses of gamma-radiation increased. We extracted sheet resistance through P^+^ TLM and analyzed by extracting concentration of holes. As a result of extracting the term in which the mobility of the hole and the concentration were multiplied according to concentration, the concentration of P^+^ increased by 2.89% and 3.12% as the doses of gamma ray increased.

**Figure 4:**
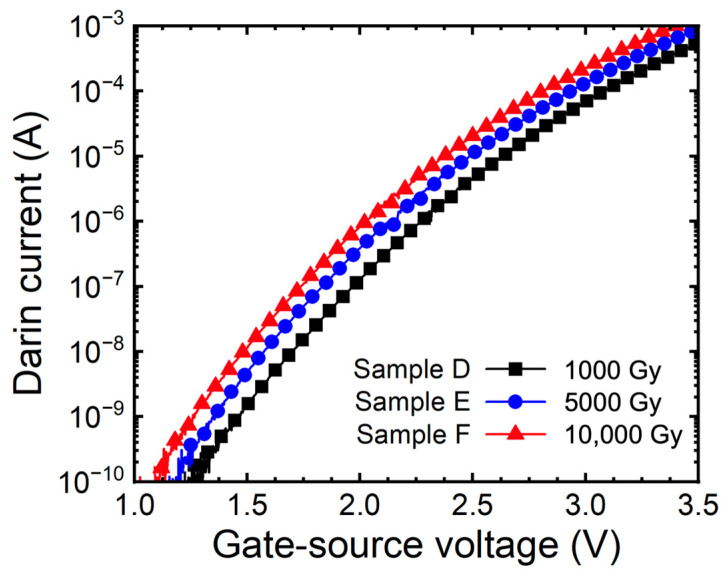
Measured sub-threshold curves (I_D_-V_GS_) of 1.2 kV SiC MOSFETs with floating state according to gamma-ray irradiation dose (1000, 5000, and 10,000 Gy).

Figure 4 depicts the sub-threshold characteristic curve of samples D, E, and F. We extracted the sub-threshold swing (SS) and verified the increase in concentration of holes [26]. As a result of the extraction, as the gamma-ray irradiation dose increased, the SS was 179 mV/dec (sample D), 185 mV/dec (sample E), and 191 mV/dec (sample F). Confirming the relationship between SS and concentration through Equation (1), V_th_ is the thermal voltage of 2.3 V, ε_Si_ is the dielectric constant of silicon and is 11.7 ε_0_, q is 1.6 × 10^−19^ C, and p is the concentration of the holes [4]. As a result, the concentration increased by 3.4% and 3.67% as the doses of gamma-ray.

(1) $$SS=(2.3)\cdot V_{th}\cdot (1+\frac{\varepsilon_{si}}{C_{ox}\sqrt{\frac{2\varepsilon_{si}\emptyset_{f}}{q\cdot p}}})$$

The C_min_ was extracted from Figure 1, where the characteristics of oxide were previously extracted, and the concentration of donors of the N-type MOS capacitor was extracted using Equation (2). ε_SiC_ is dielectric constant of SiC of 9.7 ε_0_, q is 1.6 × 10^−19^ C, ϕ_s_ is the surface electric field of semiconductor, and n is the concentration of the electrons [21]. The surface potential was extracted according to the gate voltage and the surface potential when the capacitance was at its minimum was used. Additionally, we extracted the minimum capacitance depending on the dose of gamma ray from the C-V curve in Figure 1 and observed the change in electron concentration using Equation (2).

(2) $$C_{min}=\sqrt{\frac{\varepsilon_{SiC}\cdot q\cdot n}{2\varphi_{s}}}$$

**Figure 5:**
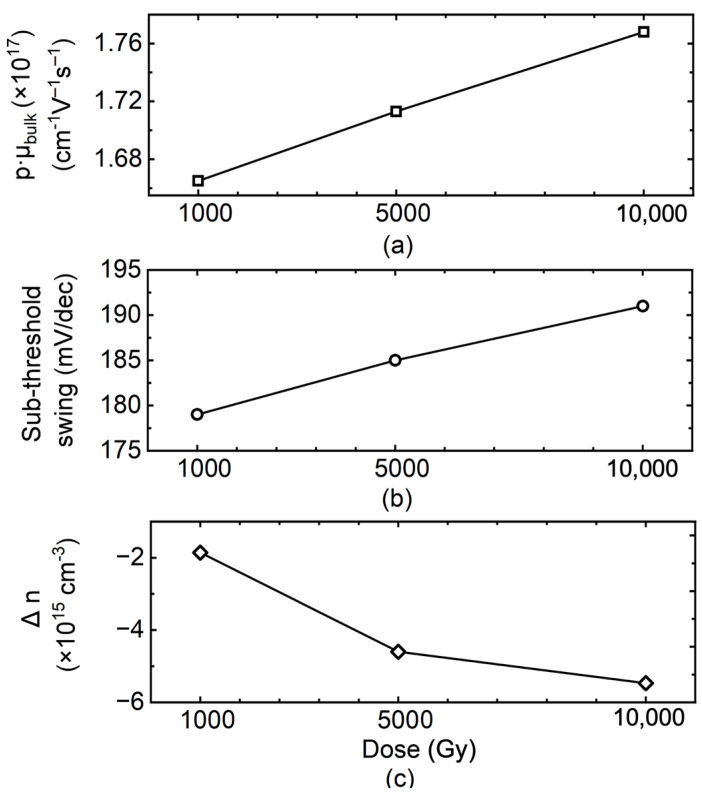
Extracted concentration of holes and electrons according to the gamma-ray irradiation dose (1000, 5000, and 10,000 Gy) from (**a**) P^+^ TLM patterns, (**b**) 1.2 kV SiC MOSFETs and (**c**) N-type SiC MOS capacitors.

As a result, as the doses of gamma-ray increased, it decreased by 2.54% (1000 Gy), 3.93% (5000 Gy), and 5.03% (10,000 Gy). Figure 5c summarizes the change in concentration of electrons according to doses of gamma-ray.

The mass action law was applied to the electron concentration to extract the hole concentration [21,27,28,29]. As a result, it was concluded that the concentration of holes increased. As a result, it was verified that the occurrence of the TID affects not only on the oxide but also on the SiC due to gamma irradiation, which is the values can be considered critical.

When SiC MOSFETs are applied in space applications, the devices are operating with biasing and in the radiation environment. So, it is needed to analyze the effects of bias conditions during gamma irradiation. We applied a voltage of +5 V to the gate of the SiC MOSFETs during gamma irradiation. The I-V and subthreshold characteristics of the SiC MOSFETs were evaluated before and after gamma irradiation. Table 1 summarizes the sample IDs of the gate bias condition experiments during gamma irradiation.

**Figure 6:**
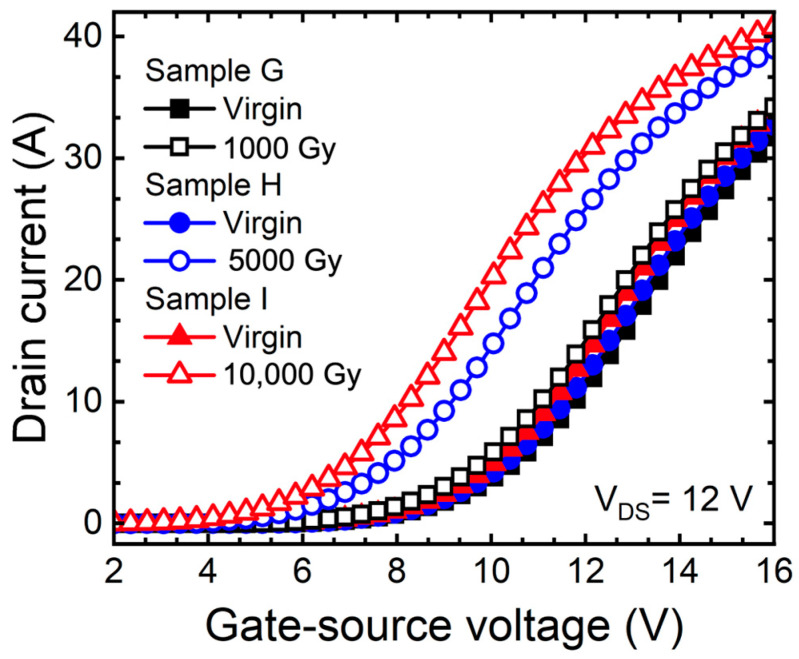
Measured transfer curves (I_D_-V_GS_) of 1.2 kV SiC MOSFETs according to gamma-ray irradiation dose (1000, 5000, and 10,000 Gy) at the bias condition V_GS_ = +5 V.

Figure 6 shows the transfer curve (I_D_-V_GS_) of the SiC MOSFETs depending on the doses of gamma-ray under gate bias conditions. The changes in characteristics were confirmed by extracting the V_T_. As a result, as the doses of gamma-ray increased, the V_T_ decreased by 1.15 V (sample G), 1.81 V (sample H), and 2.55 V (sample I). The result shows a significant difference between the unbiased SiC MOSFETs and the biased SiC MOSFETs, especially when exposed to gamma-ray. The impact of gamma-ray exposure on electrical characteristics was investigated, and it was noticed that defects generated in the oxide were responsible for the observed changes [30]. Gamma-ray is irradiated to create EHPs in the oxide, and the holes are trapped in defects. Significantly, an accelerated TID effects were observed under positive bias conditions applied to the gate during gamma irradiation [31,32]. This phenomenon was associated with the increased trapped positive charges in the generated defects, which affected the electrical characteristics.

**Figure 7:**
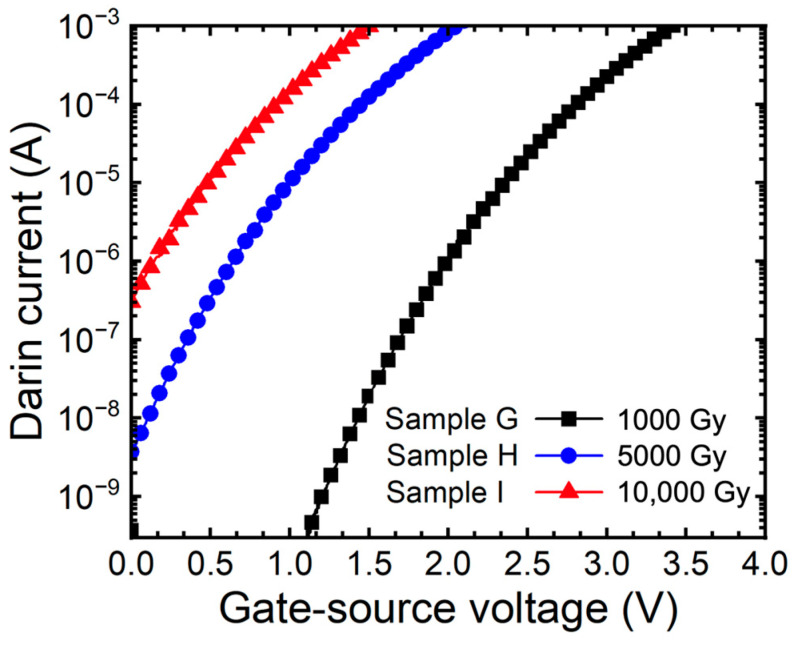
Measured sub-threshold curves (I_D_-V_GS_) of 1.2 kV SiC MOSFETs according to gamma-ray irradiation dose (1000, 5000, and 10,000 Gy) at the bias condition V_GS_ = +5 V.

Figure 7 depicts the sub-threshold curves of SiC MOSFETs under gate bias conditions. As a result of the extraction, SS is 182 mV/dec (sample G), 230 mV/dec (sample H), and 292 mV/dec (sample I), respectively. The relationship between SS and concentration of holes is analyzed, and the concentration of acceptors increased by 6.9% and 8.3% depending on the doses of gamma-ray. As a result, we noticed that irradiating gamma-ray on SiC MOSFETs under bias conditions accelerates the TID effects of SiC.

**Figure 8:**
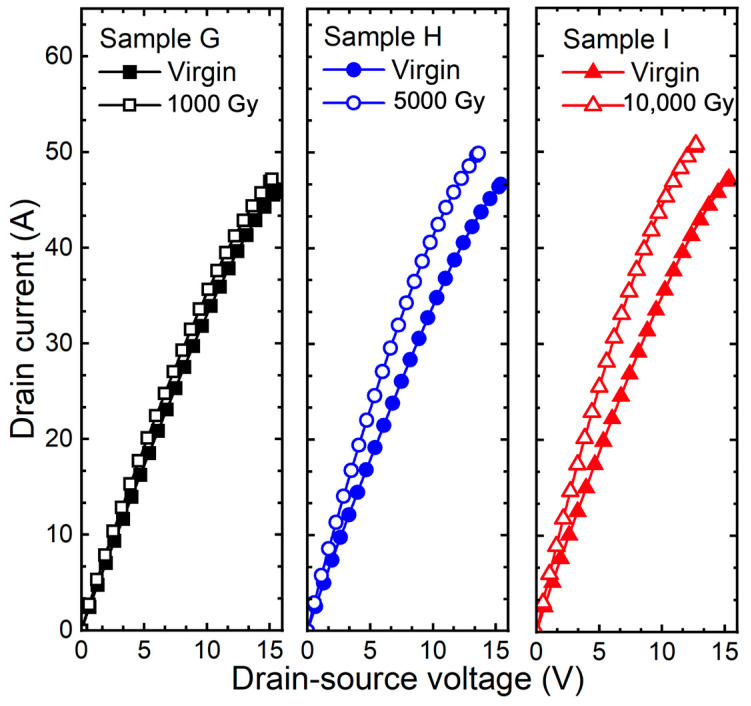
Measured output curves (I_D_-V_DS_) of 1.2 kV SiC MOSFETs according to gamma-ray irradiation dose (1000, 5000, and 10,000 Gy) at the bias condition V_GS_ = +5 V.

Figure 8 shows the output curve (I_D_-V_DS_) of 1.2 kV SiC MOSFETs applied bias according to gamma-ray irradiation doses. As a result of extracting the shift of on-resistance (ΔR_on_ = R_on,before_ − R_on,after_) is 14 mΩ (sample G), 33 mΩ (sample H), and 40 mΩ (sample I). The TID effects are accelerated by the gate bias condition, and the Q_F_ increases rapidly. This causes an accumulation of more electrons in the JFET region of the SiC MOSFETs, resulting in a decrease in R_on_ [4].

**Figure 9:**
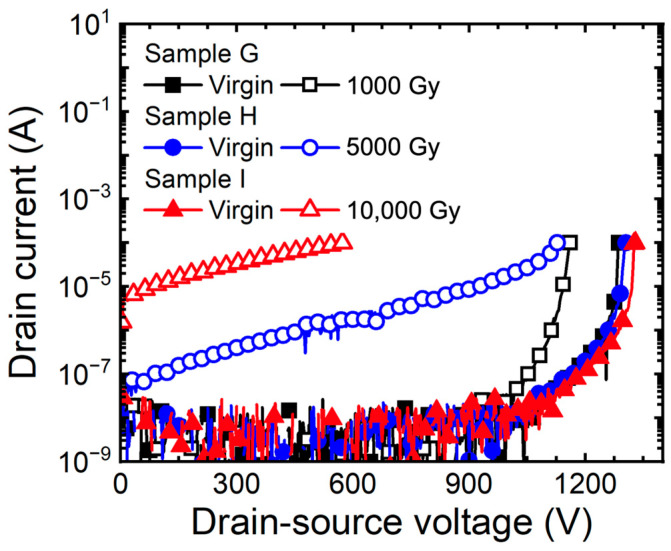
Measured breakdown characteristic curves (I_D_-V_DS_) of 1.2 kV SiC MOSFETs according to gamma-ray irradiation dose (1000, 5000, and 10,000 Gy) at the bias condition V_GS_ = +5 V.

Figure 9 depicts the breakdown characteristic curve (I_D_-V_DS_) of 1.2 kV SiC MOSFETs with applied bias according to doses of gamma-ray. As the gamma-ray irradiation dose increases, the ΔBV is 126 V (sample G), 201 V (sample H), and 771 V (sample I). The breakdown characteristic curves in Figure 9 show that punch through breakdown occurs [20]. This is caused by the rapid increase in Q_F_, which reduces the energy barrier in the channel region, resulting in punch through. Additionally, Gamma irradiation causes hole trapping in thick oxides in edge-terminated regions. Because of the effect of hole traps inside the thick oxide, it reduces the curvature of the depletion region between the main junction, the P^+^ source and the N-drift layer. The electric field becomes concentrated in the depletion region with a small curvature. The effect of the high Q_F_ causes the higher electric field to be concentrated at the main junction of the edge termination. This leads to a deterioration of the breakdown characteristics and a decrease in the breakdown voltage [33,34,35].

**Figure 10:**
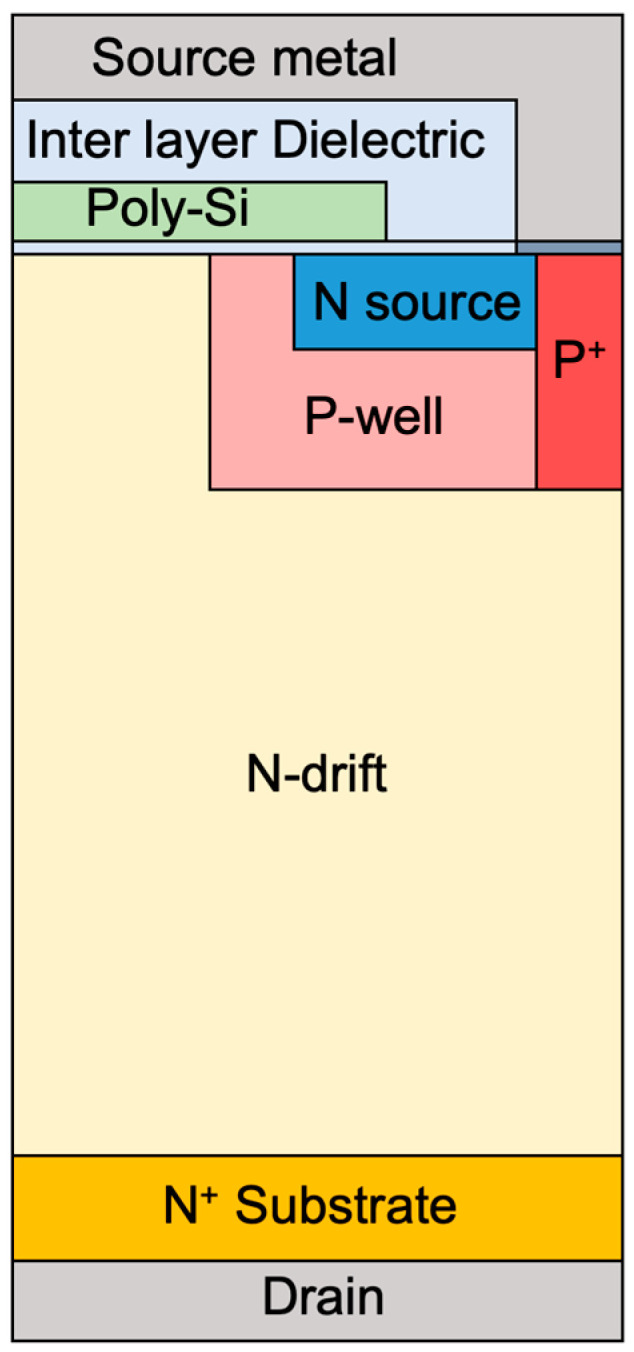
Cross-sectional view of the structure of a 1.2 kV SiC MOSFET in TCAD simulation.

When compared to the results of gamma-ray irradiation in a floating state, the overall deterioration of static characteristics can be verified to have a fatal effect on gamma-ray irradiation. Because gamma-ray was irradiated while applying a positive bias, the TID effects in the oxide is accelerated, the fixed charge in the oxide increases, and V_T_ and BV rapidly deteriorate [14,30]. We verified that TID effects are accelerated under the gate bias condition during gamma irradiation. As a result, the V_T_ of the SiC MOSFETs decreased rapidly, and punch through breakdown occurred. We used the experimental results in TCAD simulations to verify the breakdown mechanism of the SiC MOSFETs. Because the TID effects occurs throughout the SiC in the P-well, the surface concentration was extracted by changing the total concentration of P-well. Figure 10 depicts the structure with the breakdown voltage and on-resistance targeted at 1200 V and 189 mΩ in TCAD simulation. To achieve the targeted characteristics of the device, the concentration and thickness of the N-drift layer are designed to be 1 × 10^16^ cm^−^^3^ and 10 µm, respectively. The width of the JFETs on the surface of the N-drift layer is 0.7 μm with respect to the half-cell. We simulated the 1.2 kV SiC MOSFETs (Sample G, H, I) with gate bias applied by calculating the hole concentration through subthreshold swing of 1.2 kV SiC MOSFETs (Sample G, H, I) by using the increase rate of hole concentration with gate bias as a reference, because our aim was to understand the tendency of the electrical characteristics to change with the fixed charge and hole concentration, which increases with the gamma ray dose and gate voltage conditions. In addition, it is difficult to extract the fixed charge of 1.2 kV SiC MOSFET commercial devices because it is difficult to know the exact area of the oxide, therefore, we simulated the fixed charge extracted from N-type SiC MOS capacitor. The fixed charges that we obtained from the N-type SiC MOS capacitor in the floating state were 0.6 × 10^11^, 1.70 × 10^12^, and 1.94 × 10^12^ cm^−2^ for doses of 1000, 5000, and 10,000 Gy, respectively. Considering that the gate-biased device has a larger fixed charge than the device in the floating state due to the acceleration of the TID effect, we conducted simulations at conditions of 6 × 10^11^, 6 × 10^12^, 6 × 10^13^ cm^−2^. The specific design parameters of the structure we used for TCAD simulation are summarized in Table 3.

**Figure 11:**
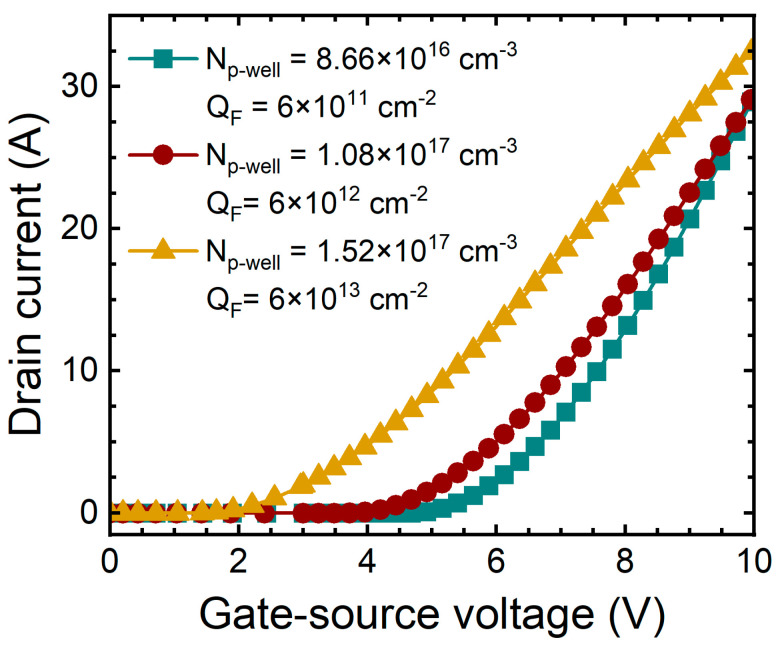
Simulated transfer curves (I_D_-V_GS_) of 1.2 kV SiC MOSFETs according to concentration of P-well and fixed charge in the oxide.

Figure 11 shows the transfer curves according to the increase in concentration of P-well and Q_F_. The extracted V_T_ is 3.93 V (device A), 3.02 V (device B), and 1.63 V (device C). The increase in Q_F_ affects the charge distribution on the SiC surface and affects V_T_. We realize that V_T_ decreases depending on the Q_F_, and these results are consistent with the behavior observed in actual SiC MOSFETs. This result shows that as Q_F_ increases, the charge distribution on the SiC surface is affected, resulting in a decrease in V_T_.

**Figure 12:**
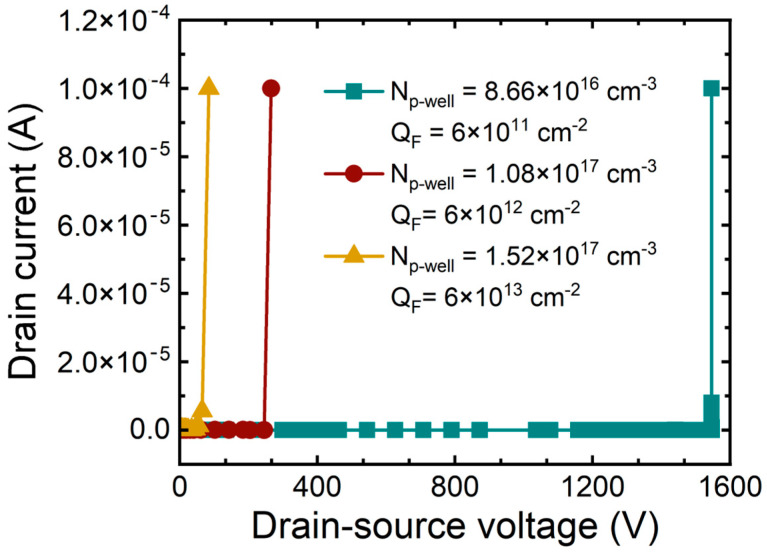
Simulated Breakdown characteristic curves of 1.2 kV SiC MOSFETs according to concentration of P-well and fixed charge in the oxide.

**Figure 13:**
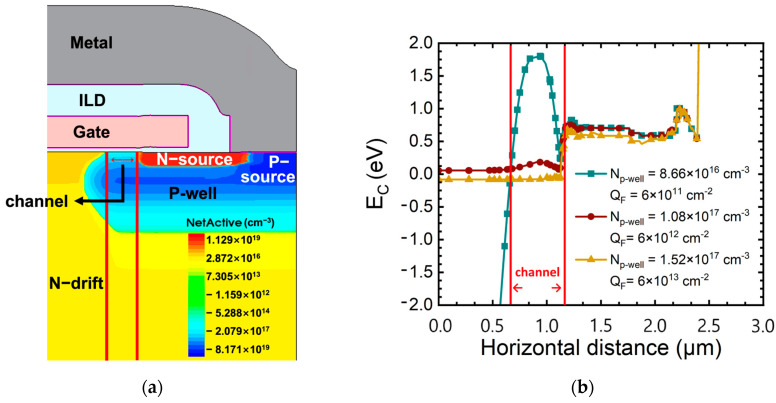
(**a**) Channel region of simulated 1.2 kV SiC MOSFET and (**b**) energy band diagram at the channel region of 1.2 kV SiC MOSFETs according to N_p-well_ and Q_F_.

Figure 12 shows the breakdown characteristic curves with increasing concentration of P-well and increasing Q_F_. The BV is 1545 V, 265 V, and 85 V, which decreased depending on the increase in concentration of P-well and Q_F_. It was noticed that as Q_F_ rapidly increases and the depletion region decreases, the breakdown characteristics deteriorate. We analyzed the energy band diagram in Figure 13 to verify the punch through breakdown mechanism observed in the experiment.

Figure 13a shows the channel region of a 1.2 kV SiC MOSFETs with the energy band diagram depicted. Figure 13b is an energy band diagram according to P-well concentration and Q_F_. We notice that the energy barrier in the channel region decreases depending on the increase in Q_F_. This accelerates the TID effects in the 1.2 kV SiC MOSFET under bias conditions, causing excessive positive charge accumulation in the oxide. This reduces the depletion region between the N-drift and P-well, lowering the energy barrier. As the barrier decreases, drain induced barrier lowering occurs, and when the barrier completely disappears, punch through breakdown occurs [21]. These simulation results agree well with the behavior observed in actual SiC MOSFETs, and it was confirmed that degradation increases as the TID effects accelerates in SiC MOSFETs to which bias is applied by gamma-ray irradiation. 

## 3. Conclusions

We analyzed the effects of bias conditions on SiC MOSFETs during gamma irradiation and performed electrical measurements and TCAD simulations to verify the degradation mechanism. The analysis showed that the V_T_ of SiC MOSFETs exposed to gamma radiation in the gate-biased condition decreased by 13% after 10,000 Gy of gamma irradiation. Moreover, the BV in the gate-biased condition decreased by 81% after 10,000 Gy of gamma irradiation, and punch through failure was observed. This is a change in electrical characteristics due to a rapid increase in Q_F_ due to acceleration of the TID effect under gate bias conditions. In addition, an increase in concentration of holes was verified through TLM and sub-threshold characteristics of SiC MOSFETs after gamma-ray irradiation, which verified the occurrence of the TID effects in SiC. Additionally, it was confirmed that the TID effects accelerated under bias conditions and the concentration of holes increased. Also, we verified the effects of Q_F_ on the punch through breakdown of the 1.2 kV SiC MOSFETs by TCAD simulation.

## Figures and Tables

**Table 1:** The gate bias conditions of SiC MOS and MOSFET samples for different doses of gamma irradiation.

Device Types	Gate Bias	Dose (Gy)	Sample ID
N-type SiC MOS capacitor	Floating state	1000	Sample A
		5000	Sample B
		10,000	Sample C
1.2 kV N-channel SiC MOSFET	Floating state	1000	Sample D
		5000	Sample E
		10,000	Sample F
	+5 V	1000	Sample G
		5000	Sample H
		10,000	Sample I

**Table 2:** Summary of oxide characteristics of N-Type SiC MOS capacitor according to doses of gamma-ray.

Sample ID	ΔV_FB_ (V)	ΔQ_F_ (cm^−2^)
Sample A	1.806	0.59 × 10^11^
Sample B	2.636	1.70 × 10^12^
Sample C	2.965	1.94 × 10^12^

**Table 3:** Sample ID by design variables and conditions for 1.2 kV SiC MOSFETs in TCAD simulation.

Cell Pitch (µm)	Channel Length (µm)	Concentration of the N-Drift Layer (cm^−3^)	Thickness of the N-Drift Layer (µm)	Width of the JFET in Half Cell (µm)	N_p-well_ (cm^−3^)	Q_F_ (cm^−2^)	Sample ID
6	0.3	1 × 10^16^	10	0.7	8.66 × 10^16^	6 × 10^11^	Device A
					1.08 × 10^17^	6 × 10^12^	Device B
					1.52 × 10^17^	6 × 10^13^	Device C

## Data Availability

Data are contained within the article.
